# Duck enteritis virus UL21 is a late gene encoding a protein that interacts with pUL16

**DOI:** 10.1186/s12917-019-2228-7

**Published:** 2020-01-08

**Authors:** Linjiang Yang, Mingshu Wang, Chunhui Zeng, Yong Shi, Anchun Cheng, Mafeng Liu, Dekang Zhu, Shun Chen, Renyong Jia, Qiao Yang, Ying Wu, Shaqiu Zhang, Xinxin Zhao, Juan Huang, Yunya Liu, Xumin Ou, Sai Mao, Yanling Yu, Ling Zhang, Bin Tian, Leichang Pan, Mujeeb Ur Rehman, Xiaoyue Chen

**Affiliations:** 1 Institute of Preventive Veterinary Medicine, Sichuan Agricultural University, Wenjiang, Chengdu City, Sichuan 611130 People’s Republic of China; 2 Key Laboratory of Animal Disease and Human Health of Sichuan Province, Sichuan Agricultural University, Wenjiang, Chengdu City, Sichuan 611130 People’s Republic of China; 3 Avian Disease Research Center, College of Veterinary Medicine, Sichuan Agricultural University, Wenjiang, Chengdu City, Sichuan 611130 People’s Republic of China

**Keywords:** Duck enteritis virus, UL21, UL16, Late gene, Interaction

## Abstract

**Background:**

pUL21 is a conserved protein of *Alphaherpesvirinae* that performs multiple important functions. The C-terminus of pUL21 in other members of this subfamily has RNA-binding ability; this domain contributes to pseudorabies virus (PRV) retrograde axonal transport in vitro and in vivo and participates in newly replicated viral DNA packaging and intracellular virus transport. However, knowledge regarding duck enteritis virus (DEV) pUL21 is limited.

**Results:**

We verified that DEV UL21 is a γ2 gene that encodes a structural protein. Moreover, we observed that pUL21 localized to the nucleus and cytoplasm. DEV pUL21 interacted with pUL16 and formed a complex in transfected human embryonic kidney (HEK) 293 T cells and DEV-infected duck embryo fibroblasts (DEFs). These results were further confirmed by CO-IP assays.

**Conclusions:**

The DEV UL21 gene is a late gene, and pUL21 localizes to the nucleus and cytoplasm. DEV UL21 is a virion component. In addition, pUL21 can interact with pUL16. These findings provide insight into the characteristics of UL21 and the interaction between pUL21 and its binding partner pUL16. Our study enhances the understanding of DEV pUL21.

## Background

Infection with duck enteritis virus (DEV), a member of the *Alphaherpesvirinae* subfamily, can cause serious clinical symptoms and pathological changes, such as vascular injury, tissue haemorrhage, gastrointestinal mucosal papulosis-like lesions, and degeneration of lymphoid and parenchymal organs [1–3]. The disease often causes severe economic losses to the global waterfowl industry [4].

The DEV genome is composed of double-stranded DNA and contains a unique long zone (UL) and a unique short zone (US) surrounded by reverse repeats at both ends of these regions [5]. UL21 is a tegument protein that is conserved among the members of *Alphaherpesvirinae* with sequence identities ranging from 27 to 84% and sequence similarities ranging from 57 to 94% [6]. However, the length of the gene encoding UL21 varies in different herpesviruses. For example, the UL21 gene in herpes simplex virus 1 (HSV-1), herpes simplex virus 2 (HSV-2), Marek’s disease virus serotype 2 (MDV-2), and DEV is 1608 bp [7], 1599 bp [8], 1596 bp [9] and 1686 bp [10], respectively. The UL21 gene in HSV-1 shows 36% similarity to that in pseudorabies virus (PRV) [11], and the UL21 gene in MDV-2 shows 29–42% similarity to that in HSV-1 [9]. In addition, the HSV-1, DEV, bovine herpesvirus 1 (BHV-1), gazelle herpesvirus 2 (GHV-2), GHV-3, PRV, equine herpesvirus 4 (EHV-4) and varicella-zoster virus (VZV) pUL21 proteins exhibit high similarity in the region comprising amino acids 73–92 [12]. The UL21 gene has been considered both a late (L) gene and an early (E)/L gene because it possesses the features of both, and its functions are related to virus particle replications, virulence, transmission and immunization [13–16]. Moreover, pUL21 contains numerous sites for modifications, such as N-glycosylation and phosphorylation [17], suggesting that the protein undergoes posttranslational modification. Studies investigating its subcellular location have shown that pUL21 is distributed in both the cytoplasm and nucleus but mainly in the former [7, 18]. Although the characteristics of many DEV genes have been reported [19, 20], the molecular properties and functions of the DEV UL21 protein have not been described to date.

In HSV-1, the presence of pUL11, pUL16 and pUL21 leads to the formation of a complex [21]. The tegument protein pUL11 is structurally related to nuclear and cellular membrane proteins and is functionally involved in the assembly and release of viral particles. pUL11 is also targeted to the Golgi apparatus, where it accumulates when expressed alone [22, 23]. pUL16 is another tegument protein associated with nucleocapsid assembly. The cysteine residues at positions 247, 269, 271, and 275 can interact with clusters of acidic amino acids and leucine motifs (AC) in pUL11. These cysteine residues also participate in the binding to residues 268–535 of pUL21 [24]. However, pUL21 and pUL11 have not been observed to interact. Studies have shown that the formation of the complex is attributed to interactions among residues 268–535 of pUL21, the first 49 residues of pUL11 and the cysteine residues at positions 247, 269, 271, and 275 of pUL16 [25]. According to the respective functions of pUL11, pUL16 and pUL21, their combined action may be related to virus assembly, release and transport. For example, pUL16 binds to the capsid prior to reaching the Golgi apparatus to promote capsid maturation. pUL11 associates with the nuclear membrane and binds to pUL16, thereby increasing the chance that pUL16 will bind to the capsid, and the capacity of pUL16 binding to the capsid is reduced by 70% in the absence of pUL11 [26, 27]. As mentioned above, pUL11 accumulates in the Golgi, and pUL21 binds to tubulin; the successful transport of the nucleocapsid to the Golgi apparatus is followed by virion budding and maturation mediated by the interaction between pUL11 and pUL21 [22, 23]. Finally, the virus is released into the extracellular environment by pUL11 [28]. pUL21, pUL11, and pUL16 are highly conserved proteins among *Alphaherpesvirinae* viruses [29]. Nonetheless, the mechanism underlying the interaction among these three proteins and the effect on the virus remain to be elucidated. In this study, we sought to determine whether this interaction occurs in DEV.

## Results

### Preparation of the DEV UL21 polyclonal antibody

**Fig. 1:**
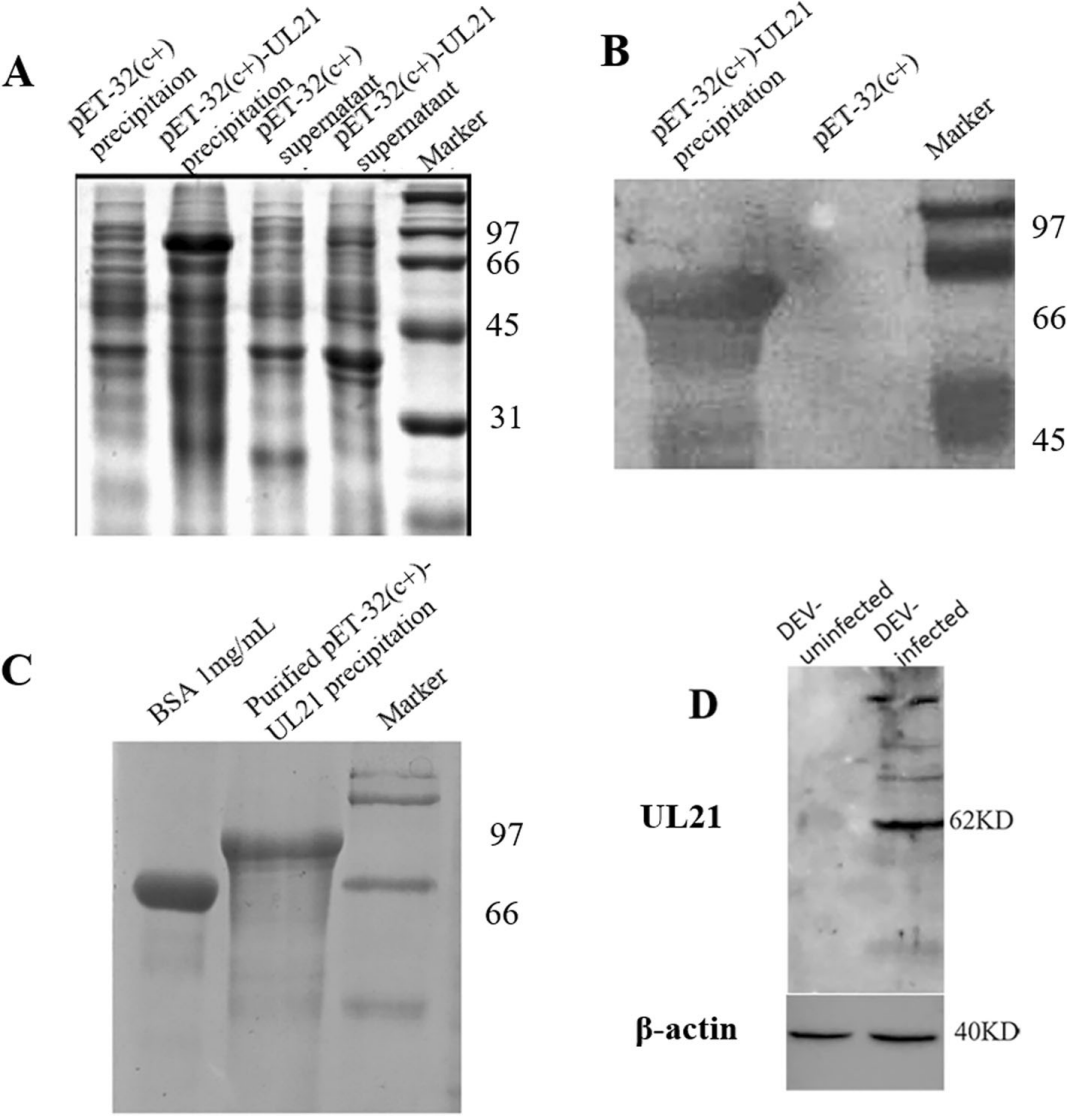
Expression, identification, and purification of recombinant UL21. **A:** Expression analysis of the recombinant protein. **B:** Immunoreactivity analysis of the recombinant protein by WB. **C:** Purification analysis of the recombinant protein. **D:** DEV UL21 was recognized by a purified polyclonal antibody

To carry out the subsequent experiment, we generated the polyclonal antibody of UL21. The UL21 gene was cloned into the vector pET-32C (+) with restriction endonuclease BamH I and Xhol I and expressed for 8 h at an induction temperature of 30 °C and a final isopropyl β-D-1-thiogalactopyranoside (IPTG) concentration of 0.4 mmol/L (Fig. 1a). The recombinant protein expressed in these cells was approximately 82 kDa in size. Rabbit anti-DEV serum was used as the primary antibody at a dilution of 1:200. The western blotting showed that the rabbit anti-DEV serum had good reactivity with pUL21 (Fig. 1b). After optimizing the conditions for pUL21 expression, gel electrophoresis and electroelution were performed to obtain products for rabbit immunization to prepare polyclonal antibodies for further study. With BSA as the reference, the purified protein concentration was 1 mg/mL (Fig. 1c). A target band of 62 kDa was obtained from infected DEFs, which is consistent with the expected size of DEV pUL21 (Fig. 1d). The results showed that the rabbit anti-UL21 antibody recognized pUL21 on the western blots.

### DEV UL21 is a late gene

**Fig. 2:**
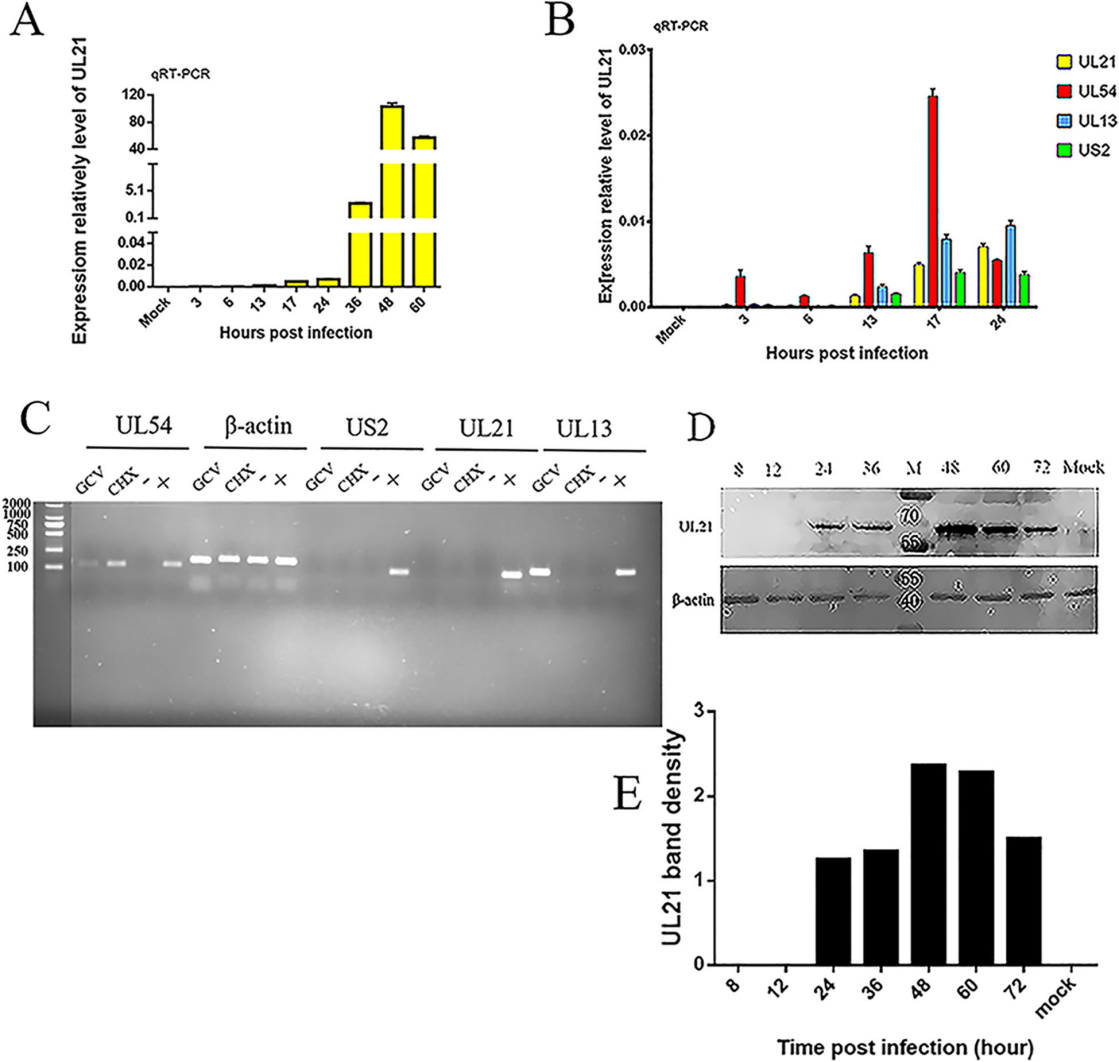
Genotype analysis of UL21 in DEV-infected cells. **A:** Transcriptional analysis of the DEV UL21 gene. The DEV UL21 gene expression data are presented as fold changes. The transcript levels of the DEV UL21 gene were normalized to those of a reference gene (β-actin). **B:** Comparison of transcriptional patterns between UL21 and the control genes UL54, UL13 and US2. **C:** The genotype of UL21 was authenticated with antiviral drug inhibition experiments. GCV represents DEV-infected cells treated with ganciclovir, and CHX represents DEV-infected cells treated with cycloheximide. (−) represents non-infected negative control cells, and (+) represents the positive control infected cells. The full image is shown in Additional file 1: Figure S1. **D:** DEV pUL21 expression. Proteins isolated from mock- or DEV-infected cells at different times were subjected to WB with anti-UL21 and anti-β-actin antibodies. **E:** A greyscale level analysis of UL21 protein expression at each time point compared with the level of β-actin protein expression

To verify the expression stage of the UL21 gene, we also investigated the expression of UL21 at different time points in infected DEF cells. UL21 transcription was first detected at 13 hpi with the highest expression level at 48 hpi (Fig. 2a). We compared the transcription pattern of the UL21 gene to the patterns of the control genes UL54, UL13 and US2 and found that the UL21 transcription level was similar to that of the L gene US2 (Fig. 2b). Subsequently, we performed a pharmacological inhibition test. We treated infected DEFs with GCV or CHX. The correct bands of the immediate E (IE) gene UL54, E gene UL13, and L gene US2 are shown; β-actin was detected as a control. However, UL21 was not detected in the negative control, GCV or CHX groups, indicating that GCV and CHX inhibited UL21 expression (Figs. 2c, Additional file 1: Figure S1). A WB analysis was performed using protein samples collected at 8, 12, 24, 36, 48, 60, and 72 hpi and a mock sample. A specific protein band of approximately 60 kDa was first detected at 24 hpi; the expression gradually increased until peaking at 48 hpi and then began to decline at 60 hpi (Fig. 2d). The band density of UL21 is shown. (Fig. 2e). These results suggest that the UL21 gene is an L gene.

### DEV UL21 localizes to both the cytoplasm and nucleus

**Fig. 3:**
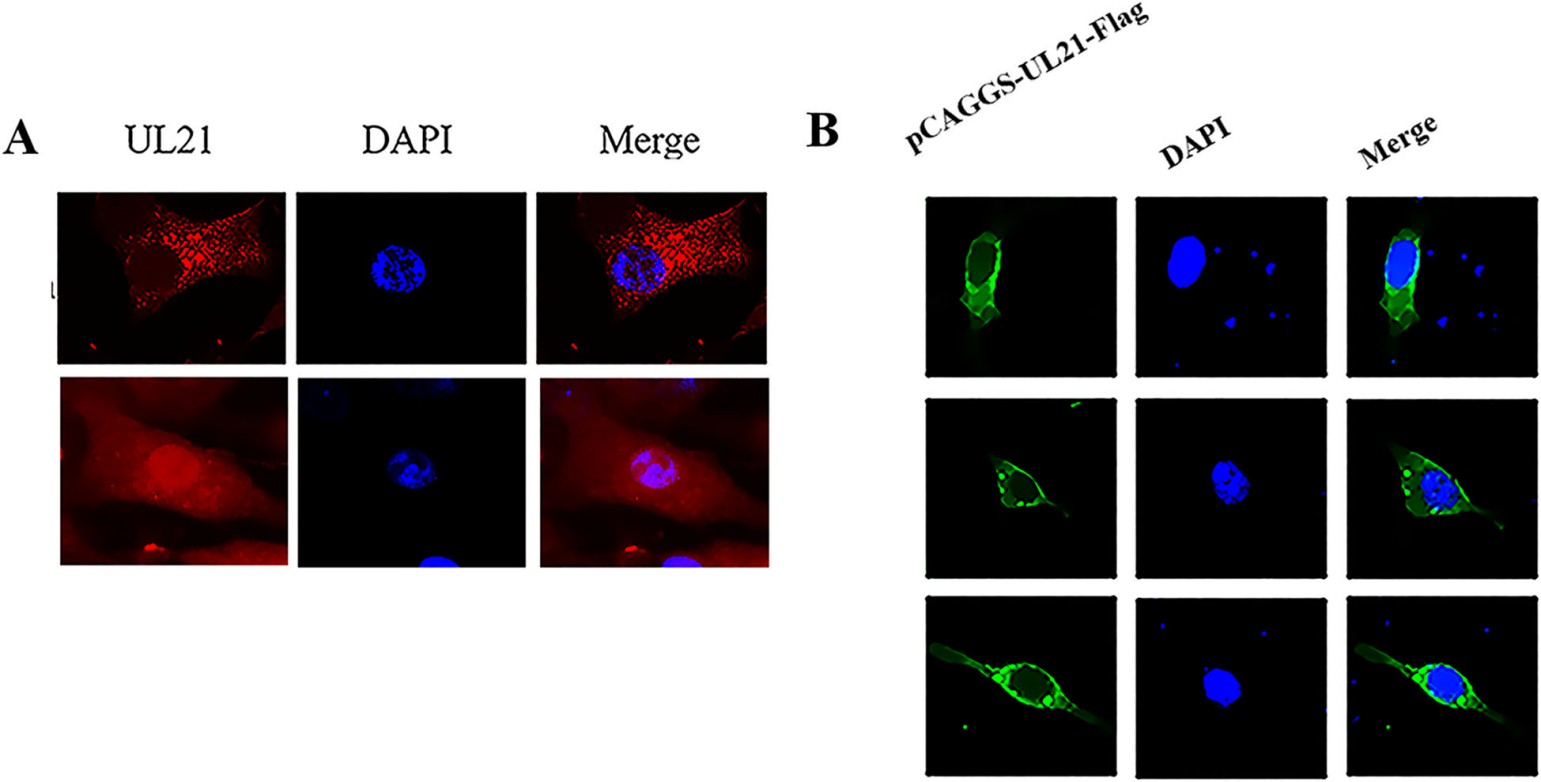
Localization of DEV UL21 in infected and transfected DEFs. **A:** DEV-infected cells on coverslips were fixed. The samples were incubated successively with rabbit anti-UL21 IgG and goat anti-rabbit IgG conjugated with Alexa Fluor 594. We captured the images by fluorescence microscopy using a 40× objective. **B:** the DEFs were transfected with pUL21 to observe localization. The samples were incubated successively with rabbit anti-UL21 IgG and goat anti-rabbit IgG conjugated with Alexa Fluor 488. The images were captured under fluorescence microscopy using a 40× objective

Previously, it was reported that pUL21 was located in the cytoplasm and nucleus. In the cells infected with 0.2 MOI of DEV, some UL21-specific staining visible as red fluorescence was distributed in the nucleus and cytoplasm. Thus, it can be concluded that UL21 is distributed in both the nucleus and cytoplasm (Fig. 3a). DEFs were also transfected with pCAGGS-UL21-Flag, the UL21 gene was cloned on a pCAGGS vector and a flag tag was added to the C-terminal of UL21; the samples were harvested at 48 h. In the cells transfected with this plasmid alone, pUL21 was distributed only in the cytoplasm (Fig. 3b).

### pUL21 is a structural protein

**Fig. 4:**
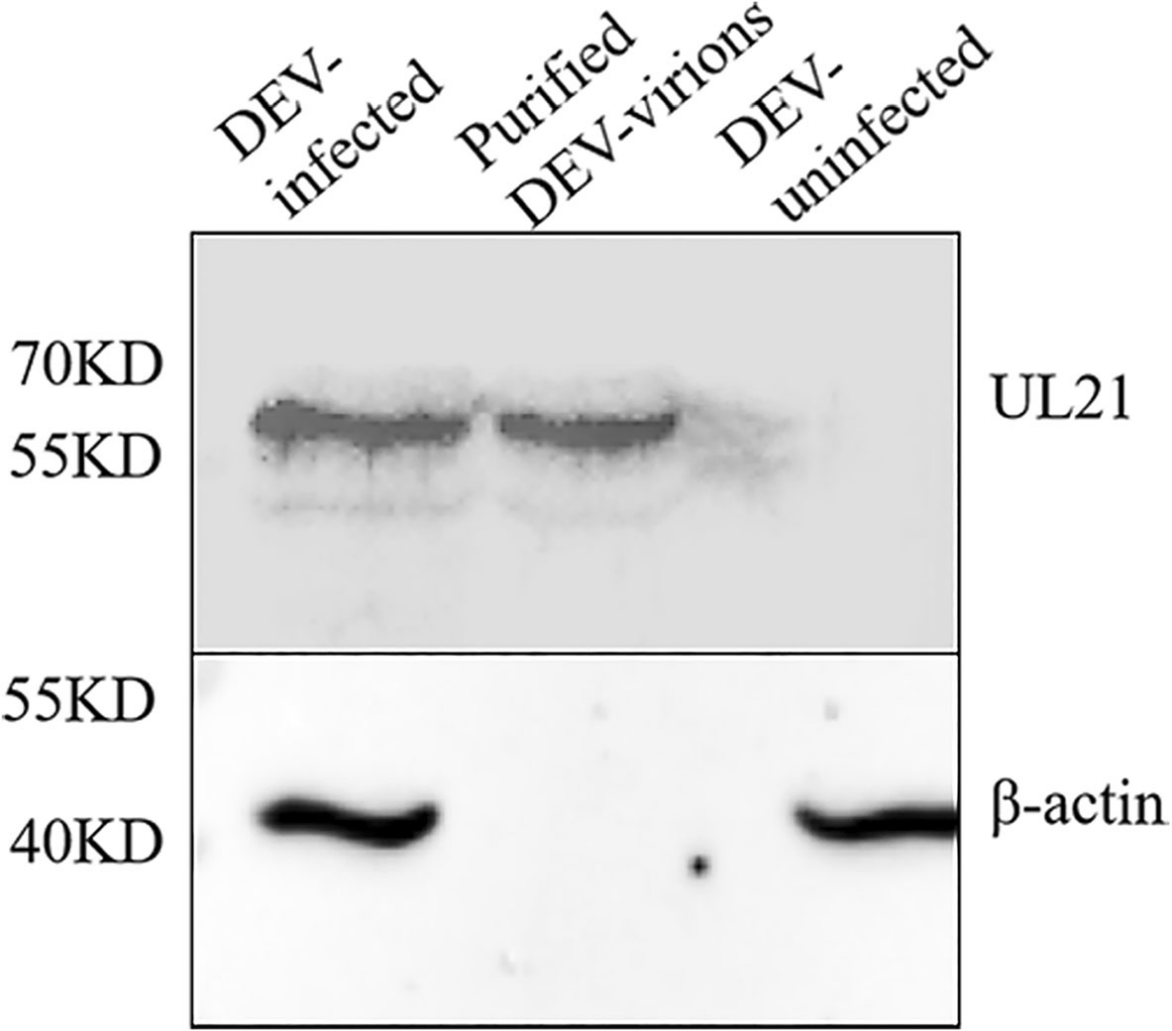
UL21 structural protein verification. Virions purified from DEF cells were separated by SDS-PAGE, transferred to PVDF membranes, and probed with antibodies against the UL21 protein and β-actin. Total mock-infected or infected cell lysates were also included as antibody controls

We evaluated the extracellular virion protein content by mass spectrometry, and the results indicated that pUL21 is present in virions. Only one unique DEV UL21 peptide was detected, and three unique peptides matched DEV gC (*P* < 0.05) (Table 1). Furthermore, we used WB to detect the purified virions, and the results were consistent with the size of pUL21 (Fig. 4). Based on the exponentially modified protein abundance index (emPAI), the relative abundance of UL21 may be low. Through the two experiments described above, pUL21 was shown to be a minor virion component.

**Table 1:** Viral content of DEV extracellular virions. gC was used as a positive control

Protein	Description	Score	Mass	Matches	Sequences	emPAI	NCBI Accession
UL44	glycoprotein C	97	47,836	6 (3)	6 (3)	0.22	AJG04885
UL41	tegument protein	46	57,546	6 (2)	6 (2)	0.12	AJG04888.1
UL21	tegument protein	109	62,752	3 (2)	2 (1)	0.05	AJG04909.1

### Localization of pUL21 and pUL16 in infected DEFs and transfected HEK 293 T cells

**Fig. 5:**
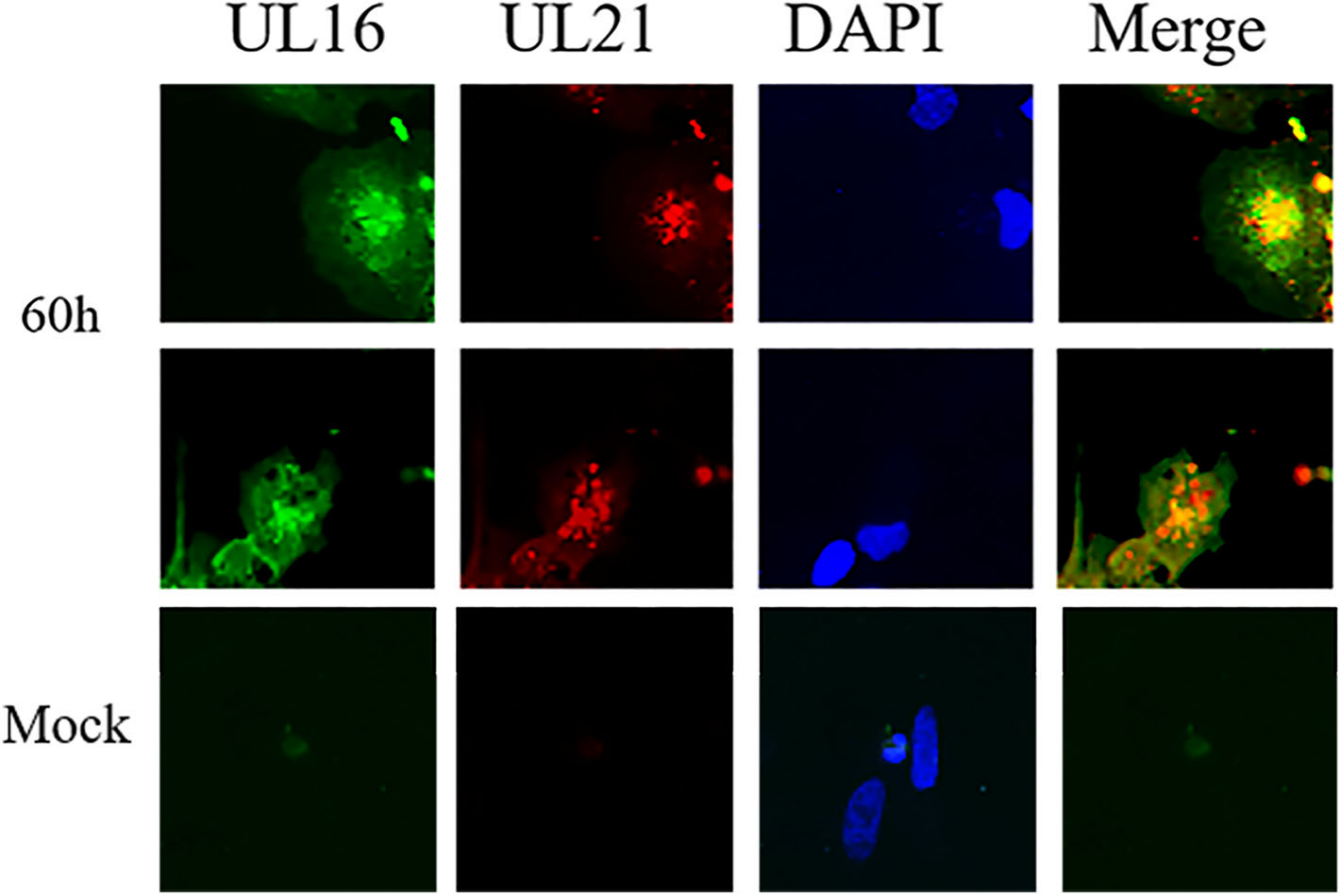
Colocalization of pUL16 and pUL21. DEV-infected cells on coverslips were fixed at 60 hpi. Colocalization of pUL16 (green) and pUL21 (red) in DEFs was assessed. The cell nuclei were stained with DAPI, and the images were captured under fluorescence microscopy using a 20× objective

If pUL21 and pUL16 interact with each other, they may be colocalized. DEFs were infected with DEV at an MOI of 0.2. The samples were collected at 60 hpi. UL21 was consistent with the previous results and distributed in the cytoplasm under the condition of DEV infection. In addition, pUL16 was distributed in the cytoplasm. Interestingly, when the images of pUL21, pUL16 and nuclear staining were merged, orange or yellow fluorescence appeared. These merged fluorescence microscopy results indicate that pUL21 and pUL16 colocalize in DEFs as the orange and yellow components represent colocalization sites (Fig. 5).

**Fig. 6:**
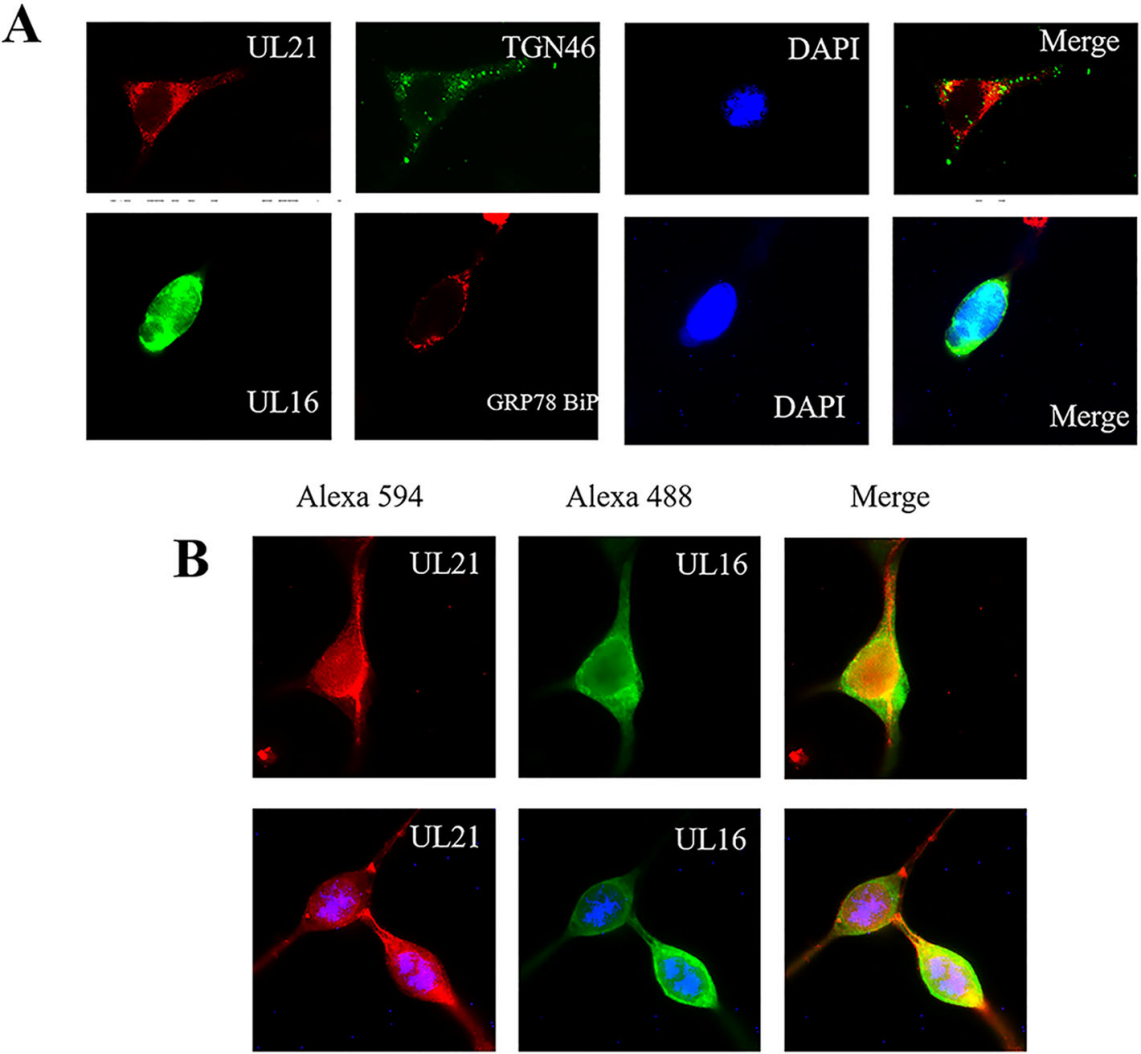
Colocalization of pUL16 and pUL21 in HEK 293 T cells. **A:** Localization of pUL21 (red) and pUL16 (green) alone with TGN46 (green) and GRP78 BiP (red) as cell markers in HEK 293 T cells. Images were captured under fluorescence microscopy using a 40× objective. **B:** Colocalization of pUL16 and pUL21 in HEK 293 T cells with pCAGGS-UL21-Flag (red) and pCMV-Myc-UL16 (green). Images were captured under fluorescence microscopy using a 40× objective

To assess whether pUL16 and pUL21 form a complex directly, plasmids encoding these proteins were transfected into HEK 293 T cells individually or together. Thus, the recombinant plasmids pCMV-Myc-UL16 and pCAGGS-UL21-Flag were transfected separately, and TGN46 and GRP78 BiP were used as cell markers (Fig. 6a). pUL21 was detected in the cytoplasm, which is consistent with Fig. 3b, whereas pUL16 accumulated in the nucleus. pUL21 colocalized with pUL16 in the co-transfected HEK 293 T cells. In contrast to the virus infection, pUL21 was distributed in the cytoplasm when transfected alone and was distributed in the nucleus and cytoplasm when co-transfected with UL16, while UL16 was distributed in the nucleus when transfected alone and distributed in both the cytoplasm and nucleus when co-transfected with UL21. After the co-transfection, yellow was observed in the nucleus and cytoplasm (Fig. 6b).

### pUL21 can interact with pUL16

**Fig. 7:**
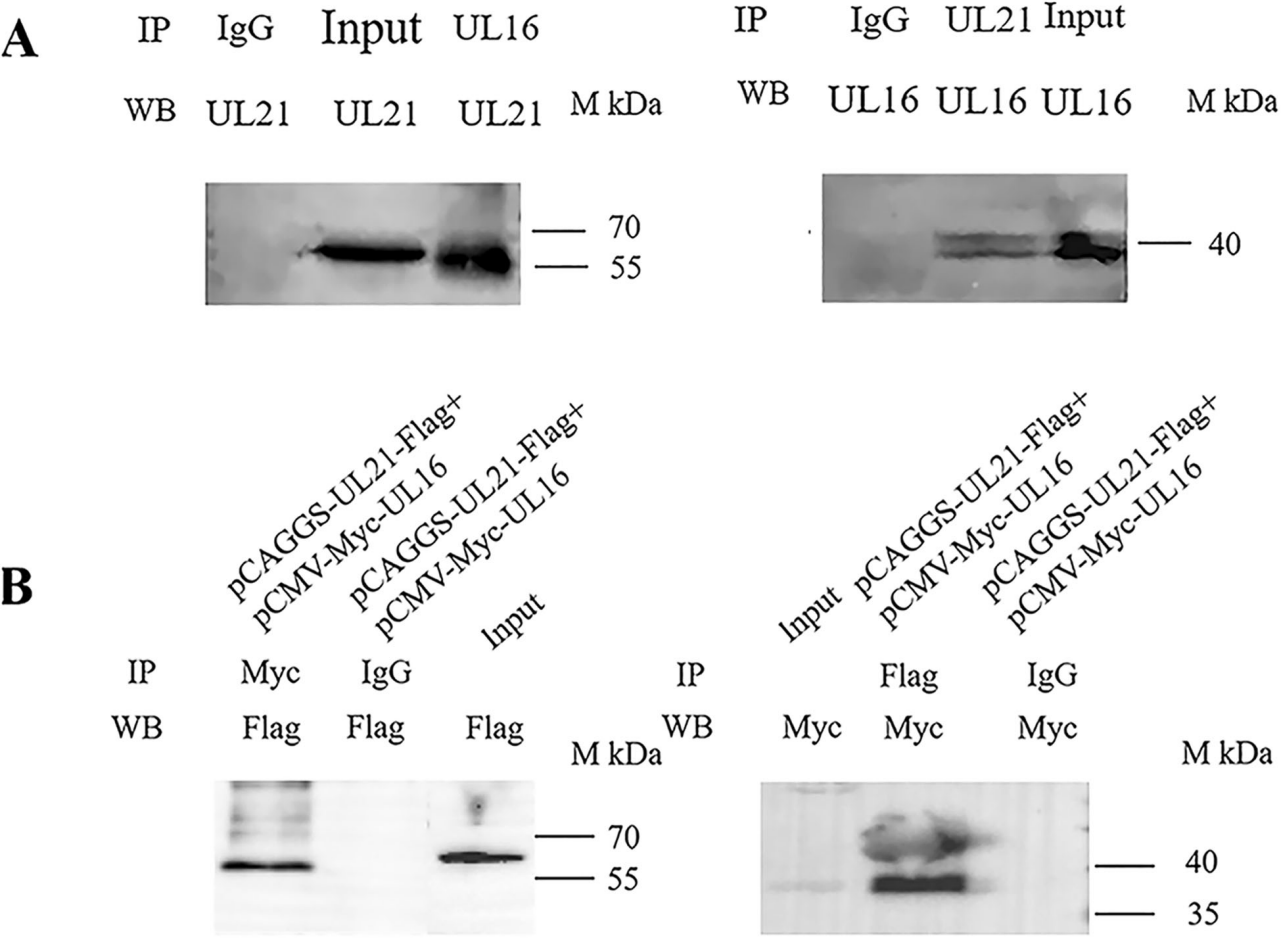
Interaction between pUL16 and pUL21. **A:** DEFs were infected with DEV. The anti-UL16 and anti-UL21 polyclonal antibodies were used for IP, and the anti-UL21 and anti-UL16 polyclonal antibodies were used for WB. **B:** HEK 293 T cells were transfected with UL21 and UL16 expression plasmids. Myc and Flag monoclonal antibodies were used for IP, and Flag and Myc polyclonal antibodies for WB

To more directly verify the interaction between the two proteins. We performed coimmunoprecipitation using infected DEFs collected at 48 hpi to verify the observed interaction. In the experimental group, pUL21 was pulled down by the rat anti-UL16 antibody and detected by the rabbit anti-UL21 antibody, revealing a band of 62 kDa (pUL21) (Fig. 7a). Similarly, in the experimental group, pUL16 was pulled down by the anti-UL21 antibody and detected by the rat anti-UL16 antibody, revealing a band of 42 kDa (pUL16) (Fig. 7a). No visible band was observed in either control group. As shown in Fig. 7b, when pUL21 and pUL16 were ectopically co-expressed in HEK 293 T cells, the anti-Myc antibody co-precipitated pUL21 with pUL16, and the anti-Flag antibody co-precipitated pUL16 with pUL21 (Fig. 7b). No visible band was observed in either control group.

## Discussion

After a herpesvirus infects a target cell, the DNA viral genome is linearized when entering the host cell nucleus, and the viral genes are transcribed through temporal cascades divided into the following three stages: the IE, E, and L stages. IE genes are expressed first, and their expression products regulate the transcription of E genes. E genes are transcribed prior to viral DNA replication, and peak expression is reached after replication begins. E genes encode proteins related to viral replication, including those that encode enzymes, such as thymidine kinase. E proteins are commonly used to regulate viral replication. L genes, which mainly encode virus structural proteins, are expressed in the L stage of transcription and require regulation by E gene products.

L genes are also called γ genes and can be divided into γ1 and γ2 genes. Although γ1 activity partially depends on viral DNA synthesis, γ2 activity requires this process [30]. For example, Baines et al. [7] treated HSV-1-infected cells with phosphate acetic acid to inhibit viral DNA replication and found that the UL21 gene of HSV-1 is an L gene. However, Mahmoudian et al. [16] used RT-qPCR to show that the infectious laryngotracheitis virus (ILTV) UL21 gene has both E and L gene properties.

To identify the DEV UL21 gene type, we used RT-qPCR and WB to study the transcription and expression kinetics of DEV UL21 at different time points in DEFs. IE genes are expressed in the presence of CHX although the transcription of E and L genes is suppressed [31]. We also treated DEV-infected cells with GCV and examined UL54 (an IE gene) [32], US2 (an L gene) [33], UL13 (an E gene) [34], and β-actin as controls. We found that UL21 gene expression was inhibited in the groups treated with GCV and CHX. We speculate that the DEV UL21 gene is a γ2 gene that strictly depends on the onset or completion of lytic DNA amplification. In recent years, numerous methods have been developed to analyse the structural proteins of herpesviruses [35, 36]; among these methods, mass spectrometry has emerged as the most widely used. Indeed, this method is very sensitive to low-abundance proteins, such as HSV-1 UL6 (12 copies of which exist in mature virus particles), and small virus proteins, such as HSV-1 US9 and UL11 (which are 90 and 96 aa long, respectively) [19, 35]. According to emPAI, DEV pUL21 is a low-abundance virion component. Notably, β-actin was also detected with the purified DEV particles possibly because it is difficult to obtain completely purified virions.

De Wind [18] reported that DEV pUL21 is distributed in the nucleus and cytoplasm of infected cells. In our study, the IFA results showed that pUL21 was mainly distributed in the cytoplasm and was rarely located in the nucleus, although small amounts could be detected around the nucleus; these results are consistent with the findings reported by De Wind [18]. However, when DEV pUL21 was expressed with pUL16 without other proteins, pUL21 was mostly localized to the nucleus, while pUL16 was mostly localized to the cytoplasm. UL16 and UL21 have been proposed to participate in DNA packaging/capsid maturation events beginning in the nucleus. For example, studies have shown that UL16 of HSV colocalizes with sites of capsid assembly [37] and that UL21 deletion mutants of PRV accumulate capsids lacking DNA [18]. Moreover, in VZV, UL16 and UL21 homologues have been found to interact with components of the DNA packaging machinery in yeast two-hybrid assays [38]. These findings indicate that pUL21 and pUL16 participate in DNA-packaging processes. However, the deletion of the HSV UL21 gene does not affect the cleavage and packaging of viral DNA [7], and whether UL16 and UL21 actually interact in the nucleus remains unknown. In our study, we found that DEV pUL21 could be transported into the nucleus when transfected with pUL16 and that pUL16 was distributed in the cytoplasm and nucleus when transfected with pUL21. Thus, it is possible that these proteins participate in DNA packaging and capsid maturation process.

HSV-1 pUL11 binds pUL16, and pUL21 can also bind pUL16; conversely, pUL21 and pUL11 do not interact with each other [11, 25, 29, 39]. pUL11 can also reduce the ability of the virus to assemble and be released [28]. pUL11 is targeted to the Golgi apparatus and accumulates in this organelle in the absence of the other proteins [22]. UL16 is a tegument protein associated with nucleocapsid assembly, and a complex is formed when the UL11, UL16 and UL21 proteins are all present. The coimmunoprecipitation data obtained in our study suggest that DEV pUL21 interacts with pUL16, which is consistent with data obtained from studies investigating PRV and HSV [11, 39]. Overall, the formation of the UL11-UL16-UL21 complex may be closely related to the transport, budding and maturation of the capsid. In fact, pUL16 promotes capsid maturation, and the microtubule structure of pUL21 and the Golgi-targeting of pUL11 are responsible for the transportation of the capsid to the Golgi apparatus, which completes the budding and maturation processes. Then, the virus is released into the extracellular environment through the action of pUL11. Further study of the functions of pUL21 and pUL16 could be helpful in elucidating the nature of DEV protein-protein interactions, and analyses of the assembly and transport of DEV particles are also warranted.

## Conclusions

DEV UL21 was determined to be an L gene localized in the cytoplasm or both the cytoplasm and nucleus. pUL21 was verified to be a structural protein given its presence in purified virus particles. pUL16 and pUL21 colocalize in the cytoplasm and nucleus in infected DEFs. Co-transfected pCMV-Myc-UL16 and pCAGGS-UL21-Flag in HEK 293 T cells also showed that pUL16 and pUL21 colocalized in the nucleus and cytoplasm. Coimmunoprecipitation confirmed that DEV pUL16 can interact with pUL21.

## Methods

### Cells and viruses

The DEV strain CHv (GenBank No. JQ647509.1) was procured from Avian Disease Research Center of Sichuan Agricultural University. Duck embryo fibroblast (DEF) cells were maintained in modified Eagle’s Minimum Essential Medium (MEM) (Thermo Fisher, USA) supplemented with 10% bovine serum at 37 °C in a 5% CO_2_ atmosphere. Human embryonic kidney (HEK) 293 T cells were maintained in Dulbecco’s Modified Eagle’s Minimum Essential Medium (Thermo Fisher, USA) supplemented with 10% foetal bovine serum (Thermo Fisher Scientific, USA), 100 U/mL penicillin and 100 μg/mL streptomycin at 37 °C in a 5% CO_2_ atmosphere.

### Antibodies and vectors

The rabbit anti-UL21 polyclonal antibodies were generated for this study, and the rat anti-UL16 polyclonal antibodies were provided by He Qin [20]. The following monoclonal antibodies were used in this study: rabbit anti-GRP78 BiP (Abcam, UK), mouse anti-TGN46 (Abcam, UK), goat anti-rabbit IgG (Thermo Fisher Scientific, USA), rabbit anti-Myc tag (Beyotime, CHN), mouse anti-Flag tag (Transgen Biotech, CHN), Alexa Fluor 594 Goat anti-Rabbit IgG (Thermo Fisher Scientific, USA), Alexa Fluor 488 goat anti-mouse IgG (Thermo Fisher Scientific, USA), Alexa Fluor 488 goat anti-rat IgG (Abcam, UK), Alexa Fluor 594 goat anti-rabbit IgG (Life Technologies, USA), and mouse anti-β-actin (Beyotime, CHN). Normal rabbit IgG was obtained from Beyotime, and normal rat IgG was obtained from Thermo. The pCAGGS [40], pCMV-Myc [41], and pET-32c plasmids were provided by the Sichuan Agricultural University Avian Diseases Research Center.

### Preparation and identification of polyclonal antibodies

pUL21 was expressed and purified via gel and electric elution. Approximately 1 mg of UL21 was emulsified in complete Freund’s adjuvant (Sigma, GER) and used to immunize rabbits through intradermal injections. Subsequent booster doses of 1 mg, 1.5 mg and 0.5 mg were prepared in incomplete Freund’s adjuvant, and the protein was administered after 2 and 3 weeks by subcutaneous injection. To collect the antibodies, the rabbits were bled through an ear vein 1 week after the final immunization. The antiserum was harvested, and preliminary purification was conducted using saturated ammonium sulfate. The antibody production followed the Sigma polyclonal antibody production method.

### Western blotting

For the western blotting (WB), lysates were separated by SDS-PAGE, and then, the proteins were transferred to a polyvinylidene difluoride (PVDF) membrane (Millipore, MA, USA), which was subsequently blocked with blocking buffer (5% skim milk and 0.1% Tween 20 in PBS) for 1 h at room temperature. The membrane was incubated overnight at 4 °C with rat anti-UL16 or rabbit anti-UL21 monoclonal antibodies at dilutions of 1:100 or rabbit anti-Myc or mouse anti-Flag polyclonal antibodies at dilutions of 1:1000. Then, the membrane was washed three times with PBST and incubated with HRP-conjugated goat anti-rabbit IgG, goat anti-mouse IgG or goat anti-rat IgG (1:3000) secondary antibodies for 1 h at 37 °C. The membrane was washed three times with PBST, and the signals were developed using an enhanced chemiluminescence (ECL) kit (Takara, JPN).

### Quantitative reverse transcription PCR

The total RNA was isolated from the DEV-infected DEFs at different time points (3, 6, 13, 17, 24, 36, 48 and 60 hpi), and then, reverse transcription was performed; an uninfected control was included. The primers were designed with Oligo 7 (Additional file 1: Table S1). Quantitative reverse transcription PCR was performed in a 20-μL reaction volume containing 10 μL of SYBR Green mix (Takara, JPN), 1 μL of each primer, 1 μL of cDNA, and 7 μL of RNase-free water. Triplicate experiments were performed to analyse UL21, UL54, UL13, US2 and β-actin gene expression, and the relative transcription levels were calculated using the 2^-ΔCt^ method [26].

### Pharmacological inhibition

Pharmacological inhibition was performed to confirm the DEV UL21 gene expression patterns. Three flasks of DEFs were prepared and inoculated with DEV as follows: one flask was prepared without any drugs, and the other flasks contained either 300 μg/mL ganciclovir (GCV, a DNA polymerase synthesis inhibitor) or 100 μg/mL cycloheximide (CHX, a protein synthesis inhibitor). The total RNA was isolated from the DEV-infected DEFs incubated with GCV or CHX (Meilunbio, CHN) at 24 hpi and subsequently reverse-transcribed into cDNA. Then, the cDNA was used for a PCR analysis.

### Immunofluorescence analysis

Cells grown on coverslips were washed three times with PBS and fixed overnight with 4% paraformaldehyde in PBS at 4 °C. For the indirect immunofluorescence analysis (IFA), the fixed cells were permeabilized with 1% Triton X-100 in PBS for 30 min at 4 °C and incubated with 200 μL of blocking buffer (3% bovine serum albumin in PBS) in a humidified chamber for 1 h at 37 °C. Then, the cells were incubated with primary antibodies (rabbit anti-UL21 and rat anti-UL16 at a dilution of 1:200) and Alexa Fluor-conjugated secondary antibodies (at a dilution of 1:1000) in blocking buffer for 60 min at 37 °C. The samples were examined under a Nikon H550L fluorescence microscope.

### Transfection

The cells were transfected at 90 to 95% confluence with 2.5 μg of plasmid DNA added to 125 μL of MEM and mixed well; then, 3.75 μL of Lipofectamine 3000 (Thermo Fisher Scientific, USA) in 125 μL of MEM were added, and the cells were gently mixed and incubated at room temperature for 5 min. The DNA suspension and 4 μL of p3000 were mixed together and incubated at room temperature for 15 min, and then, the mixture was added to a 6-well plate. The plate was shaken gently and placed in a 37 °C cell incubator.

### Coimmunoprecipitation

The DEFs were infected with the DEV strain CHv at a multiplicity of infection (MOI) of 0.2. The infected DEFs were washed twice with cold PBST, and PMSF was added to the immunoprecipitation (IP) cell lysis buffer (Beyotime, CHN) at a final concentration of 1 mM. Precooled IP cell lysis buffer at 100 μL/mL was added to the cells, which were scraped from the plates, placed on ice, and shaken slowly on a horizontal shaker for 15 min until they were fully lysed. The cells were centrifuged at 14,000×g for 15 min at 4 °C, and the supernatant was collected. Protein A + G agarose (Bio-Rad, USA) was washed three times with PBST. Rat anti-UL16 IgG and rabbit anti-UL21 IgG (rat anti-UL16 and rabbit anti-UL21 monoclonal antibodies at dilutions of 1:10) were added to the agarose beads. Rabbit anti-Myc or mouse anti-Flag polyclonal antibodies were also used at dilutions of 1:100. The samples were gently rotated at room temperature for 30 min. Then, the complexes were washed three times with PBST. The lysates containing the target proteins were added, and the mixture was incubated at 4 °C overnight under gentle rotation. The samples were washed with PBST, the complexes were rapidly centrifuged for 30 s, and the supernatants were collected. Finally, 1 × SDS loading buffer was added, and the samples were heated for 10 min at 70 °C.

### Mass spectrometry

SDS-PAGE was used to separate the purified virion samples. The products were stained with Coomassie brilliant blue (Bio-Rad, USA) and then sent to Sangon Biotech Company (Sangon Biotech, CHN) for a liquid chromatography-tandem mass spectrometry (LC-MS/MS) analysis. The methods used for the in-gel trypsin digestion, LC-MS/MS, and database searches have been described in detail by Loret et al. [35].

### Virion purification

The DEFs were infected with the DEV strain CHv at an MOI of 5. At 2 hpi, the cells were washed twice with PBS, and the medium was replaced with Opti-MEM. At 72 hpi, the medium was collected and clarified by centrifugation at 2000×g for 20 min at 4 °C to remove the cell debris. The DEV virions were harvested by ultracentrifugation (40,000×g, 2 h, 4 °C) through a 30% (wt/vol) sucrose cushion and then banded by isopycnic gradient ultracentrifugation in a continuous 30 to 60% (wt/vol) potassium tartrate gradient in TBS (40,000×g, 2 h, 4 °C). The band containing the virions was collected, diluted tenfold in TBS, and pelleted by ultracentrifugation (20,000×g, 30 min, 4 °C). The pellets were resuspended in TBS and stored at − 80 °C [19].

## Supplementary information

**Additional file 1: Table S1.** Sequence and characteristics of RT-qPCR primers. The primers of DEV UL21, UL54, UL13, US2, β-actin were designed with Oligo 7. **Figure S1.** Complete image of UL21 genotype identification. GCV represents DEV-infected cells adding ganciclovir and CHX is adding cycloheximide. The (−) represents negative control and (+) were positive control.

## Data Availability

The datasets used and/or analyzed during the current study are available from the corresponding author on reasonable request.

## Abbreviations

BHV-1: Bovine herpesvirus 1
CHX: Cycloheximide
DAPI: 4′,6-diamidino-2-phenylindole
DEF: Duck embryo fibroblasts
DEV: Duck enteritis virus
EHV-4: Equine herpesvirus-4
emPAI: Exponentially modified protein abundance index
GCV: Ganciclovir
GHV-2: Gazelle herpesvirus 1
HEK 293 T cells: Human embryonic kidney 293 T cells
HSV: Herpes simplex virus
ILTV: Infectious laryngotracheitis virus
MDV-2: Marek’s disease virus serotype 2
NLS: Nuclear localization signal
PRV: Pseudorabies virus
PVDF: Polyvinylidene fluoride
RT-PCR: Real-time quantitative reverse-transcription PCR
VZV: Varicella-zoster virus
WB: Western blotting
