# Supplementary material for: Duck enteritis virus UL21 is a late gene encoding a protein that interacts with pUL16
Source: BMC Vet Res. 2020 Jan 8;16:8. doi: 10.1186/s12917-019-2228-7 (PMC6950997; doi:10.1186/s12917-019-2228-7)
Supplement: Supplementary file 1 — Additional file 1: Table S1. Sequence and characteristics of RT-qPCR primers. The primers of DEV UL21, UL54, UL13, US2, β-actin were designed with Oligo 7. Figure S1. Complete image of UL21 genotype identification. GCV represents DEV-infected cells adding ganciclovir and CHX is adding cycloheximide. The (−) represents negative control and (+) were positive control. [file 12917_2019_2228_MOESM1_ESM.docx]

Supplementary Material

Duck enteritis virus UL21 is a late gene and encodes a protein that interacts with pUL16

**Linjiang Yang ^1,2,3¶^, Mingshu Wang^1,2,3¶^, Chunhui Zeng^1,2^, Yong Shi, Anchun Cheng^1,2,3*^, Mafeng Liu^1,2,3^, Dekang Zhu^2,3^, Shun Chen^1,2,3^, Renyong Jia^1,2,3^, Qiao Yang^1,2,3^, Ying Wu^1,2,3^, Shaqiu Zhang^1,2,3^,Xin-Xin Zhao^1,2,3^, Yunya Liu^1,2,3^, Yanling Yu^1,2,3^, Ling Zhang^1,2,3^ , Bin Tian^1,3^,Leichang Pan****^1,3^, Mujeeb Ur Rehman^1,3^, Xiaoyue Chen^1,2,3^, Juan Huang^1,2,3^**

*** Correspondence:** Anchun Cheng: chenganchun@vip.163.com

# Supplementary Figures and Tables

# Table 1. Sequence and characteristics of RT-qPCR primers.

| Primer | Primer sequence (5’-3’) | Gene | Product size (bp) |
| --- | --- | --- | --- |
| P1 | TACGCCAACACGGTGCTG | β-actin | 178 |
| P2 | GATTCATCATACTCCTGCTTGCT |  |  |
| P3 | GCCCAGGAACACCAGTCT | DEV UL21 | 106 |
| P4 | CAGTGCGTATTGCCGTCT |  |  |
| P5 | GCCACCAACCCTACCAAG | DEV UL13 | 131 |
| P6 | GTCGTCAGCCCATCACCA |  |  |
| P7 | AGACGGTTCCGA-AAGTACAG | DEV US2 | 111 |
| P8 | TCGGCAGCACCAATAATCC |  |  |
| P9 | GAACAACCGCCGAACAC | DEV UL54 | 127 |
| P10 | TCAAACATCCGCCTCAA |  |  |


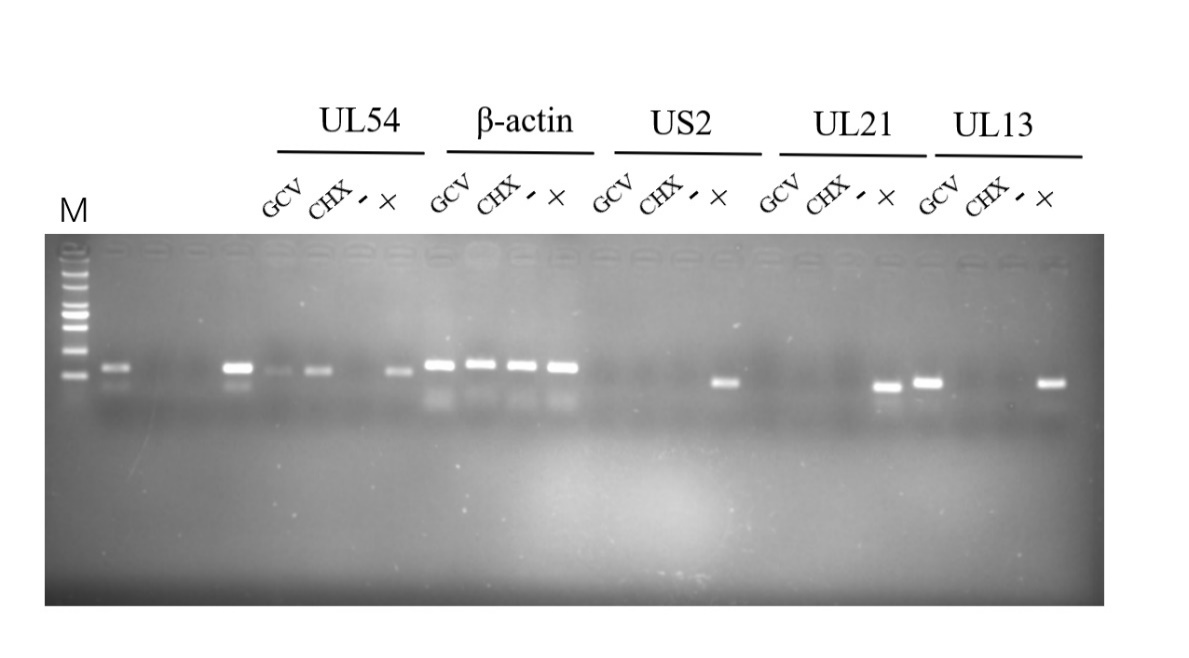


**Figure 1.** Complete image of UL21 genotype identification. GCV represents DEV-infected cells adding ganciclovir and CHX is adding cycloheximide. The (-) represents negative control and (+) were positive control.
